# Recovering Image Quality in Low-Dose Pediatric Renal Scintigraphy Using Deep Learning

**DOI:** 10.3390/jimaging11030088

**Published:** 2025-03-19

**Authors:** Marta Arsénio, Ricardo Vigário, Ana M. Mota

**Affiliations:** 1 Physics Department, NOVA School of Science and Technology, NOVA University of Lisbon, 2829-516 Caparica, Portugal; m.arsenio@campus.fct.unl.pt (M.A.); r.vigario@fct.unl.pt (R.V.); 2 Laboratory for Instrumentation Biomedical Engineering and Radiation Physis, NOVA University of Lisbon, 2829-516 Caparica, Portugal; 3 Instituto de Biofísica e Engenharia Biomédica, Faculdade de Ciências, Universidade de Lisboa, 1749-016 Lisboa, Portugal

**Keywords:** ^99m^Tc-MAG3, deep learning, noise reduction, pediatric renal scintigraphy, medical imaging

## Abstract

The objective of this study is to propose an advanced image enhancement strategy to address the challenge of reducing radiation doses in pediatric renal scintigraphy. Data from a public dynamic renal scintigraphy database were used. Based on noisier images, four denoising neural networks (DnCNN, UDnCNN, DUDnCNN, and AttnGAN) were evaluated. To evaluate the quality of the noise reduction, with minimal detail loss, the kidney signal-to-noise ratio (SNR) and multiscale structural similarity (MS-SSIM) were used. Although all the networks reduced noise, UDnCNN achieved the best balance between SNR and MS-SSIM, leading to the most notable improvements in image quality. In clinical practice, 100% of the acquired data are summed to produce the final image. To simulate the dose reduction, we summed only 50%, simulating a proportional decrease in radiation. The proposed deep-learning approach for image enhancement ensured that half of all the frames acquired may yield results that are comparable to those of the complete dataset, suggesting that it is feasible to reduce patients’ exposure to radiation. This study demonstrates that the neural networks evaluated can markedly improve the renal scintigraphic image quality, facilitating high-quality imaging with lower radiation doses, which will benefit the pediatric population considerably.

## 1. Introduction

In recent decades, imaging techniques have been consistently used for the determination and analysis of pediatric kidney diseases. There are several widely applied renal imaging modalities, including computed tomography (CT), ultrasound (US), magnetic resonance imaging (MRI) and nuclear medicine [1,2,3].

As is well known, CT and US techniques only provide anatomical information. Magnetic resonance imaging can also provide functional information in addition to anatomical (fRMI); however, its use in the renal context is still limited due to the lack of standard protocols [4,5]. Nuclear medicine techniques, such as positron emission tomography (PET) and single-photon emission computed tomography (SPECT), offer functional information, but require longer acquisition times, which can be a challenge for pediatric patients due to the need for prolonged immobility. On the other hand, scintigraphy is a simpler and faster 2D technique. As it significantly reduces the examination time compared to PET or SPECT, it is preferred for children [6,7].

Children with suspected renal pathologies often undergo several medical imaging exams, the frequency of which depends on the severity of the disease and the response to treatment [8]. Approximately 90% of pediatric radionuclide studies are focused on non-oncological pathologies, with approximately half focused on renal studies, a trend that has remained stable in developed countries [9]. Early diagnosis and treatment of urinary abnormalities can reduce comorbidities and infant mortality [10].

The increase in medical imaging exams in recent years has raised concerns about cumulative radiation exposure and cancer risk [11]. Some patients receive high doses, with one in one hundred twenty-five exposed to more than 50 mSv on a single CT examination and three in ten thousand exposed to more than 100 mSv in a single day [12]. A study in the United Kingdom revealed that 0. 68% of the patients received more than 100 mSv in 5.5 years, increasing their cancer risk [13]. Children are especially vulnerable, with a higher lifetime risk of cancer due to radiation [11,14].

For pediatric nuclear medicine, determining the correct amount of activity to administer depends on a variety of factors specific to the child being imaged. This includes the reason for the acquisition, any variations in physiology, as well as the size and shape of the patient [15,16].

According to the Society of Nuclear Medicine and Molecular Imaging guidelines, the injected radiation in ^99m^Tc-MAG3 scintigraphy examinations should be 3.7 MBq/kg (0.10 mCi/kg) [17]. In pediatric imaging, the lower radiation dose leads to fewer photon interactions within the body. This results in fewer detected photons, reducing the overall signal. As a consequence, the image noise increases. The higher noise level degrades the clarity of anatomical structures, complicating diagnostic interpretation. Therefore, optimizing the image quality is crucial, especially in pediatric patients, where minimizing radiation exposure is a priority [18].

Several approaches have been explored to reduce radiation doses while maintaining image quality. Hsiao et al. [19] evaluated the minimum dose of ^99m^Tc-MAG3 required for pediatric renal scintigraphy without compromising the diagnostic quality or renal function quantification. Starting from an initial dose of 7.4 MBq/kg, they simulated reductions to 50%, 30%, 20%, and 10%. Four doctors assessed the images with and without noise reduction. The study found that doses could be reduced to 30% without affecting the image quality, and a 20% reduction caused a slight degradation, corrected by noise reduction. At 10%, the image quality dropped significantly, though the renal function remained stable. With noise reduction, the dose could be decreased to 1.5 MBq/kg without noticeable quality loss, and accurate renal function quantification was achieved, even at 0.74 MBq/kg. However, the image evaluation depended on subjective assessments by physicians, and the study did not explore fully automated noise reduction approaches. Nagayama et al. [20] examined dose reduction in pediatric CT by lowering the tube voltage and using iterative reconstruction (IR). The low voltage reduced the radiation level and improved the contrast with iodinated media, although it increased the noise, which IR mitigated. These techniques maintained diagnostic quality. Although IR proved to be effective, it can be computationally demanding and is not directly applicable to scintigraphy. Studies by Miglioretti and Journy [21] demonstrated that reducing the highest pediatric CT doses can lower cancer risk. Automatic voltage selection systems, like CARE kV, enable up to 50% dose reduction without the loss of diagnostic quality. Feghali et al. [22] developed the S-Vue™ scoring system to assess image quality in pediatric X-rays. They showed that a 50% dose reduction across different areas and weight categories maintained satisfactory image quality. Their study, involving six hundred ninety-six digital radiographs, used four dose levels (100%, 50%, 32%, and 12.5%) processed with S-Vue™, and image quality was evaluated using the intraclass correlation coefficient (ICC). However, this approach was designed for X-ray imaging and does not extend to modalities like scintigraphy, where noise characteristics differ. Minarik et al. [23] used convolutional neural networks (CNNs) to reduce Poisson noise in whole-body bone scintigraphy, comparing their results to those obtained using traditional filters. CNNs, trained on 40,000 image pairs, reduced noise by 92%, outperforming standard filters (88%). CNNs improved the mean-squared error and noise reduction without compromising image quality or metastasis detection. The study concluded that CNNs could halve the scan time while maintaining diagnostic accuracy. Nevertheless, their work focused on whole-body bone scintigraphy rather than specific-organ imaging, such as pediatric renal scintigraphy.

Despite these advancements, several challenges remain unaddressed. Prior studies have demonstrated that dose reduction is feasible, but many relied on subjective image evaluations rather than objective automated approaches [19]. Although iterative reconstruction has proven to be effective in dose reduction for CT imaging, its computational demands and lack of direct applicability to scintigraphy limit its practical use [20]. Additionally, methodologies developed for other imaging modalities, such as X-ray imaging, do not necessarily translate to scintigraphy because of differences in noise characteristics [22]. Although CNNs have been successfully applied to noise reduction in whole-body scintigraphy, their impact on specific-organ imaging, such as pediatric renal scintigraphy, remains unexplored [23]. Addressing these gaps is crucial, as pediatric patients are particularly sensitive to radiation exposure, making dose optimization a priority in nuclear medicine. This study introduces a novel deep-learning-based approach tailored to pediatric renal scintigraphy, leveraging CNNs to enhance image quality while enabling significant dose reduction. Utilizing a publicly available dataset and objectively evaluating multiple state-of-the-art networks, this work establishes a reproducible and scalable framework for improving diagnostic imaging in vulnerable populations.

In this paper, we analyzed the applications of different deep convolutional neural networks (CNNs) specifically designed for image denoising in pediatric renal scintigraphy. Four popular networks were considered: DnCNN, UDnCNN, DUDnCNN, and an attentional generative adversarial network (AttnGAN), using images from a publicly available database. The final image quality was assessed through the SNR for the kidneys and the MS-SSIM between the original data (without dose reduction) and the denoised data.

To the best of our knowledge, this is the first study to address dose reduction in pediatric renal scintigraphy by improving image quality using CNNs in a publicly accessible dataset. This transparency not only fosters reproducibility but also fills a gap in the literature, as most related studies rely on proprietary clinical datasets. Additionally, the combination of a pediatric focus, dose reduction, and deep learning represents a novel approach for improving diagnostic imaging. By simulating dose reduction through subsampled image summation, this study offers a practical method to assess noise reduction strategies. Moreover, the comparative evaluation of four established networks provides a robust benchmark for future research. These findings emphasize the potential of CNNs to support safer imaging protocols while maintaining diagnostic quality, especially in vulnerable populations, such as pediatric patients.

## 2. Materials and Methods

### 2.1. Database

This study used the Public Database of Dynamic Renal Scintigraphy [24], specifically the drsbru set, which contains 100 dynamic renal studies with ^99m^Tc-MAG3 in children aged from 0 to 17 years. Each case consisted of a DICOM 3D set of images, consisting of 120 frames at 128 × 128 pixels, captured every 10 s for 20 min. All the ^99m^Tc-MAG3 renograms were performed at University Hospital St. Pierre following EANM guidelines [25]. To simulate reduced doses, the acquired data (100% of the counts) were divided into subsets representing 50% of the original counts. This approach aimed to demonstrate that these reduced subsets can sufficiently represent the full data while preserving the image quality, compared to that obtained using the total administered dose.

Table 1 provides a detailed summary of the selected dataset. The average injected activity per patient was not available in the database. However, an assumption based on ^99m^Tc-MAG3 can be mode using average weight values for children aged from 0 to 17, according to the 50th percentile.

Because the database contains clinical images from a wide age range, cases were carefully selected. The 100 cases were divided into one-year age groups, with one case chosen from each group. Dose reduction was simulated by reducing the summed data to 50%, corresponding to proportional reductions in the simulated dose.

### 2.2. Deep-Learning-Based Approach

Scintigraphic images are primarily affected by Poisson noise because of the inherent statistical nature of radioactive decay. This noise model leads to fluctuations in pixel intensities, particularly in low-count regions, which can degrade image quality and affect clinical interpretation. Given the statistical properties of Poisson noise, deep-learning-based denoising approaches must effectively distinguish signals from noise while preserving anatomical structures. The approach for the denoising of pediatric scintigraphic images with less dose involved using deep-learning algorithms implemented with specific software and hardware. MATLAB R2024b was used for metric analysis, while Python 3.12.4 was used to train the neural networks. The algorithms were run on a MacBook Air (2017) with a 1.8 GHz Intel Core i5 processor and 8 GB of RAM. The goal was to train deep-learning models to reduce noise and improve image quality. Despite advancements in nuclear medicine, applying deep learning to reduce noise in low-dose images remains exploratory. Our approach involved training four existing architectures from scratch: DnCNN, UDnCNN, DUDnCNN, and AttnGAN [26,27]. These architectures were chosen because of their proven capability in removing structured and unstructured noises while maintaining image fidelity.

#### 2.2.1. Denoising Convolutional Neural Network (DnCNN)

**Figure 1:**
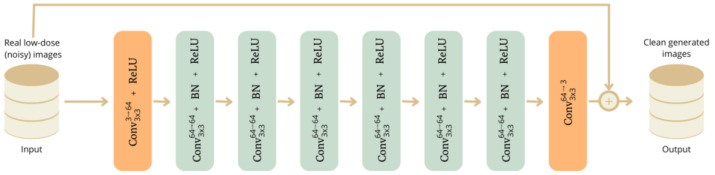
Schematic of a DnCNN. The input is 128 × 128 × 120 images.

In the first stage, a DnCNN was implemented to reduce noise in scintigraphic images. This network consists of eight convolutional layers, and a simplified schematic of its architecture is shown in Figure 1.

The network begins with an input convolutional layer using a 3 × 3 kernel (the window size most typically used in this context) and 64 filters (the basic configuration of the implementation used), which processes the 3D image and generates 64 feature maps.

Next, six convolutional layers (the basic configuration of the implementation used) with 3 × 3 kernels and sixty-four filters are applied, each followed by batch normalization (BN) and a rectified linear unit (ReLU) activation. This deepens feature extraction while progressively reducing noise.

The final convolutional layer uses a 3 × 3 kernel with 3 filters, producing a three-channel output image that matches the original. This architecture is designed to reduce noise, using padding to preserve image dimensions and BN to optimize and speed up the training. The absence of pooling layers ensures image details are preserved, which is crucial for effective noise removal.

#### 2.2.2. Unsupervised Denoising Convolutional Neural Network (UDnCNN)

**Figure 2:**
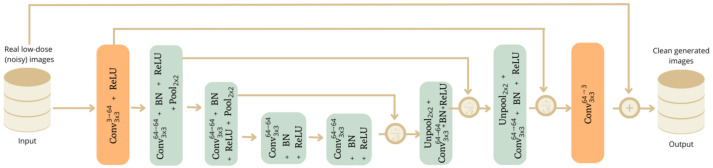
Schematic of a UDnCNN. The input is 128 × 128 × 120 images.

The UDnCNN is an enhanced version of the DnCNN, designed to improve noise reduction using a U-shaped architecture. As shown in Figure 2, this structure combines contraction and expansion paths, similar to those of U-Net, enabling the capture of both local and global image features.

The numbers of filters in the DnCNN and UDnCNN were chosen based on empirical results from prior studies and optimization experiments conducted during model development. The use of 64 filters in the initial layers ensures a rich feature extraction process, capturing both low- and high-frequency components. As the network progresses, filters maintain their dimensionality to preserve feature consistency throughout the contraction and expansion paths. The final layer reduces the feature maps to three channels, restoring the image to its original format while ensuring the optimal noise suppression.

In the contraction path, the network applies successive 3 × 3 convolutions, followed by ReLU activations and 2 × 2 max pooling (the value commonly employed in this scenario) operations, storing the indices. This strategy reduces spatial resolution while increasing the receptive field, enabling the identification of high-level image features.

To restore the original resolution, the expansion path uses 2 × 2 unpooling (the commonly referenced value in this setting) operations, recovering spatial dimensions based on the stored indices. After unpooling, additional convolutions reconstruct the image at its original resolution, integrating detailed features from the contraction phase. These features are combined by summing them and normalizing the result by dividing by $\sqrt{2}$, ensuring that the scale of the activations remains consistent across layers.

The network begins with a convolutional layer of sixty-four filters, followed by six additional convolutional layers, all with sixty-four filters and padding to maintain the image size. The final layer reduces the feature maps to three channels, corresponding to the original image.

This combination of contraction and expansion allows the UDnCNN to capture both fine details and global information, making it an efficient architecture for noise reduction in images.

#### 2.2.3. Dual-Domain Unsupervised Denoising Convolutional Neural Network (DUDnCNN)

**Figure 3:**
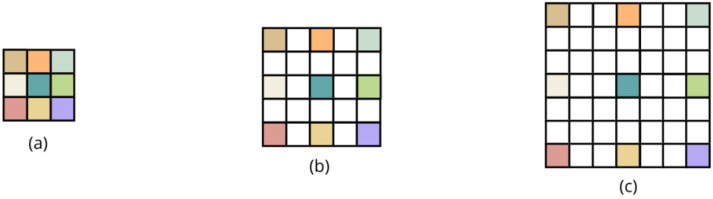
Dilated convolutions applied to the DUDnCNN architecture. (**a**) represents a 3 × 3 convolutional kernel with a dilation of 1; (**b**) represents a 3 × 3 kernel dilated by a dilation of 2, i.e., a 5 × 5 kernel with “holes” of size 1; (**c**) represents a 3 × 3 kernel dilated with a dilation of 3, i.e., a 7 × 7 kernel with “holes” of size 2.

The DUDnCNN is an improved version of the UDnCNN, designed to reduce noise by combining dilated convolutions with a U-shaped structure inspired by U-Net networks. The main modification is the introduction of dilated convolutions, which increase the receptive field without requiring pooling. This is crucial in image restoration tasks, like noise reduction, where preserving the precise location of the details is essential. Although its structure is similar to that of the UDnCNN (see Figure 2), the DUDnCNN replaces pooling and unpooling operations with dilated convolutions that have a dilation factor of 2 (the basic configuration of the implementation used), as shown in Figure 3.

The network begins with a convolutional layer that transforms the three-channel input image (RGB) to sixty-four feature channels. In the contraction path, 3 × 3 dilated convolutions are applied, followed by ReLU activations. The dilation increases progressively along the convolutional layers, adjusted based on pooling (κ) and unpooling (ι) values, with each dilated convolution following the formula $2^{\left(k-l\right)}-1$. This design enables the network to capture high-level image features while preserving important spatial details.

Unlike traditional architectures, which reduce spatial dimensions after each convolution, the DUDnCNN expands the receptive field using dilated filters. To avoid increasing the number of parameters, the filter is adjusted by inserting “spaces” between its rows and columns, as illustrated in Figure 3. This approach enhances the receptive field without compromising the computational efficiency or the network’s ability to generalize.

During the spatial resolution recovery phase, the dilated convolutional layers adjust the dilation factor in reverse, eliminating the need for unpooling.

The DUDnCNN architecture includes several convolutional layers with 64 filters, using padding to keep the image size constant. The final convolutional layer reduces the feature maps to three channels, corresponding to the original image, completing the restoration process.

#### 2.2.4. Attention Generative Adversarial Network (AttnGAN)

**Figure 4:**
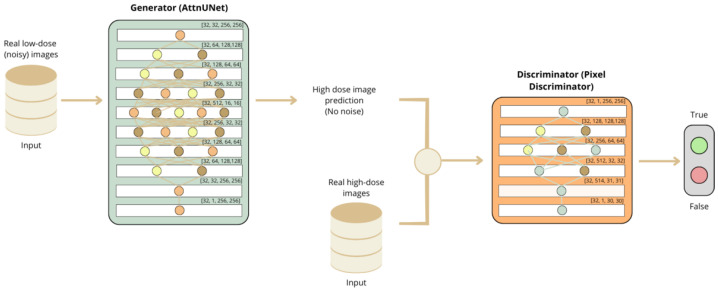
Schematic of a GAN for low-dose images.

Recent methods for noise reduction in images have used GAN networks, leveraging advanced neural architectures to enhance image quality by removing noise while preserving essential details. To tackle this challenge, we propose using an AttnGAN network, which efficiently and accurately performs noise reduction. The general structure of the AttnGAN is illustrated in Figure 4 and consists of two main components: the generator and the discriminator.

The generator, termed AttnUNet, utilizes a sequence of convolutional and subsampling layers, followed by upsampling layers and additional convolutions to restore the image to its original form. Initially, it applies a convolutional layer to the input image (single channel), resulting in 64 feature maps. The generator then performs four downsampling steps that reduce the spatial dimensions while increasing the feature depth, as illustrated in Figure 4. Each downsampling stage combines convolution and max pooling operations, followed by a convolutional block attention module (CBAM) to enhance the capture of important features.

Following the downsampling phase, the upsampling process begins, consisting of four layers that progressively recover the spatial dimensions while reducing the feature depth. Each upsampling layer includes a transposed convolution, convolutions, and CBAMs, as shown in Figure 4. The final convolutional layer adjusts the output dimensions to [32, 1, 256, 256], where 32 represents the batch size, 1 is the number of channels, and 256 × 256 corresponds to the spatial resolution of the reconstructed image.

The discriminator, also known as *PixelDiscriminator*, differentiates between real images and those generated by the generator. It features a repetitive structure of convolutional layers with varying feature depths, as depicted in Figure 4. The last layer reduces the spatial dimension of the image to [32, 1, 30, 30], yielding the final noise-reduced image.

This combination of the generator and discriminator enables the AttnGAN to effectively reduce noise, ensuring a faithful reconstruction of the original image while preserving important details and eliminating unwanted artifacts.

### 2.3. Image Analysis

The image quality was assessed using the signal-to-noise ratio (SNR) and the multiscale structural similarity index (MS-SSIM) between the original and filtered data.

The SNR is a key indicator of image quality, measuring the prominence of a signal relative to the background noise. It was calculated using Equation (1), where $\mu_{L}$ and $\mu_{B}$ represent the mean pixel values in the regions of interest (ROIs) for the lesion and background, respectively, and $\sigma_{B}$ is the standard deviation of the background ROI.

(1) $$SNR=\frac{\mu_{L}-\mu_{B}}{\sigma_{B}}$$

**Figure 5:**
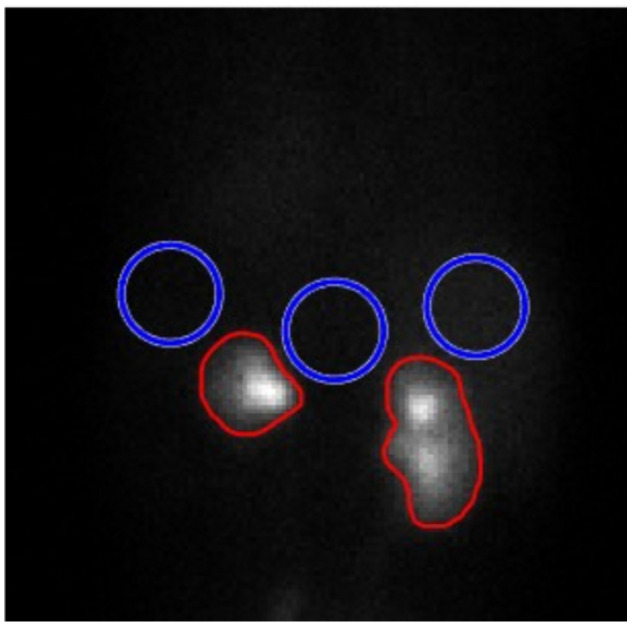
Example of the ROIs considered in this study. Red: areas of increased renal activity. Blue: background.

The mean size of the lesion, represented by the kidney regions (marked in red), was calculated, along with the mean and standard deviation of the background in the surrounding areas (marked in blue), as illustrated in Figure 5, which shows two examples of the drawn ROIs.

Because the SNR can be measured quickly and easily, it is a traditional and widely used metric for assessing noise reduction. The MS-SSIM measures the similarity between two images at multiple scales, providing a comprehensive evaluation of the image quality by capturing details at different resolutions.

The MS-SSIM calculation (Equation (2)) involves three main components: intensity, contrast, and structure. These components are weighted and combined to reflect the overall similarity between the images.

(2) $$MS-SSIM\left(x,y\right)={\left[l_{M}\left(x,y\right)\right]}^{\alpha_{M}}\cdot \prod_{j=1}^{M}{\left[c_{j}\left(x,y\right)\right]}^{\beta_{j}}\cdot {\left[s_{j}\left(x,y\right)\right]}^{\gamma_{j}}$$

where $l_{M}\left(x,y\right)$ represents the luminance component between images x and y, $c_{j}\left(x,y\right)$ denotes the contrast component at scale j, and $s_{j}\left(x,y\right)$ reflects the structural similarity at scale j. The parameters $\alpha_{M}$, $\beta_{j}$, and $\gamma_{j}$ define the relative importance of the luminance, contrast, and structure components, respectively. Finally, *M* represents the number of scales used in the MS-SSIM calculation.

The MS-SSIM values range from 0 to 1, with 1 indicating perfect similarity.

The performance differences among the different networks were tested using a *t*-test (for normal distributions) and a Wilcoxon signed-rank test (for non-normal distributions), with a two-tailed *p*-value of <0.05 indicating a significant difference.

### 2.4. Training Options

From the original 100 pediatric renal scintigraphic images acquired at 100% data counts, additional images were generated by reducing the counts to 50%, resulting in a total of 200 paired images. Before training, all the images were preprocessed to ensure consistency in input dimensions and intensity normalization. Each image was resized to 128 × 128 pixels, and pixel intensity values were normalized to a range between 0 and 1. Data augmentation techniques were not applied, as the dataset already included paired images reflecting different noise levels (100% and 50% data counts), which inherently provided a variety of noise patterns for training.

Of the 200 paired images, 134 pairs (67%) were used for training, 32 pairs (16%) were set aside for validation during training, and the remaining 34 pairs (17%) were reserved for testing the model. The mean-squared error (MSE) was selected as the loss function because it effectively minimizes the average squared difference between the predicted and original images, aligning with the goal of reducing the pixel-level noise while preserving the structural integrity.

The hyperparameter tuning involved several stages. Initially, the CNNs were trained at three learning rates: $1.0\times 10^{-3}$, $1.0\times 10^{-4}$, and $1.0\times 10^{-5}$. The optimal learning rate was determined by evaluating the balance between the signal-to-noise ratio (SNR) and the multiscale structural similarity index measure (MS-SSIM), as detailed in the Image Analysis section. Next, the batch size was varied among four, sixteen, and thirty-two to assess the impacts on the models’ performances, also using the SNR and MS-SSIM as metrics. After identifying the best learning rate and batch size, the stochastic gradient descent (SGD) optimizer was compared with Adam, and Adam was ultimately selected for its superior performance in terms of the convergence speed and stability, as described in Section 2.3.

Next, the batch size was adjusted to four, sixteen, and thirty-two, evaluating the models’ performances based on the SNR and MS-SSIM. After determining the optimal configuration, the performance of the stochastic gradient descent (SGD) optimizer was compared with that of Adam.

To ensure the robustness of the chosen hyperparameters, the average relative error for the 17 test patients was calculated, as shown in Equation (3). This relative error quantifies the difference between the model-generated images and the original images, normalized by the higher intensity between the two as follows:

(3) $$\mathrm{Relative}\;\mathrm{Error}=\frac{\mathrm{Neuronal}\;\mathrm{Network}\;\mathrm{Image}\;-\;\mathrm{Original}\;\mathrm{Image}}{\max(\mathrm{Neuronal}\;\mathrm{Network}\;\mathrm{Image},\;\mathrm{Original}\;\mathrm{Image})}$$

These steps ensured a comprehensive exploration of the training process, optimizing the model’s ability to denoise pediatric scintigraphic images effectively.

## 3. Results

**Figure 6:**
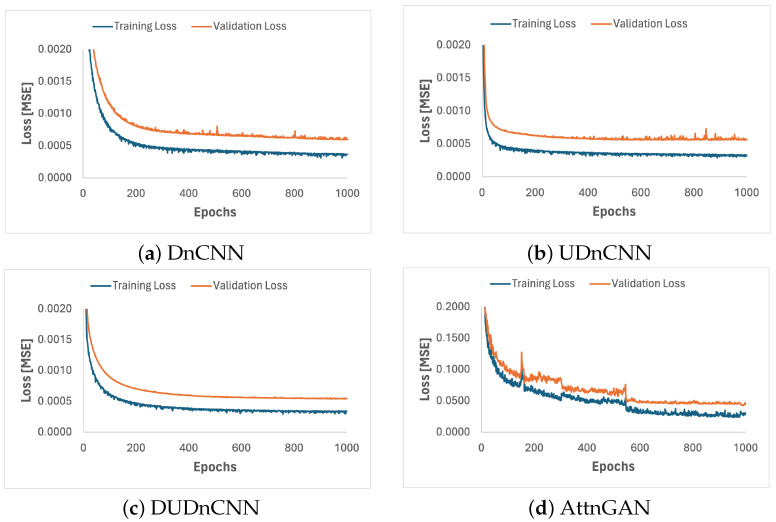
Training progress of the DnCNN (**a**), UDnCNN (**b**), DUDnCNN (**c**) and AttnGAN (**d**). Plots of the training and validation losses (MSEs) per epoch. Epoch 0 corresponds to the initial loss for the training and validation sets, before applying the network.

Four neural networks were implemented: DnCNN, UDnCNN, DUDnCNN, and AttnGAN. The training progress of these networks is shown in Figure 6.

To determine the optimal hyperparameters for the neural networks, models were trained multiple times with various configurations. The training ran for 1000 epochs, as the validation loss stabilized in the final 200 epochs, with variations remaining under 4%. This stability indicates that the models reached a plateau, where further improvements would be minimal. Additionally, there was no divergence between the training and validation curves, reducing the risk of overfitting.

Table 2 summarizes the training settings used to optimize the hyperparameters, including the batch size, learning rate, and optimizer.

The best hyperparameters—the learning rate, batch size, and optimizer—are presented in Table 3, according to the percentage of the relative error between the original image and the images generated by each of the four networks.

The results obtained from the four networks were analyzed using the optimal hyperparameters identified earlier. The SNR values for the right and left kidneys, along with the MS-SSIM, were compared across the networks. Both quantitative and qualitative approaches were used to assess the image quality.

**Figure 7:**
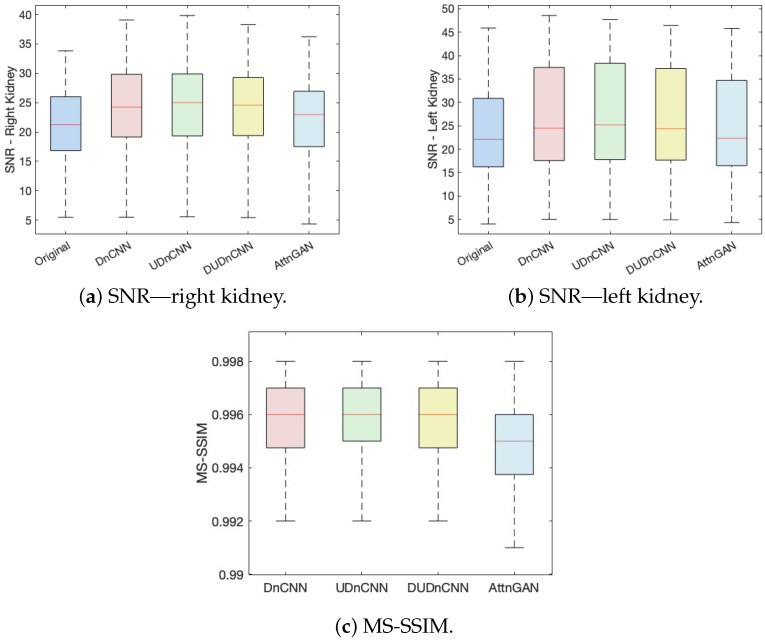
Comparison of the SNRs of the right (**a**) and left kidneys (**b**) and the MS-SSIMs (**c**) of the different architectures.

Figure 7 presents three box plots comparing the performances of the different neural network architectures by considering the SNRs of both the right and left kidneys and the MS-SSIM.

The gains (+) or losses (−) of the SNRs and MS-SSIMs for the different processing methods—DnCNN, UDnCNN, DUDnCNN, and AttnGAN—are shown in Table 4. These values, calculated based on the percentage of the relative error, indicate that the UDnCNN outperforms the other neural networks.

**Figure 8:**
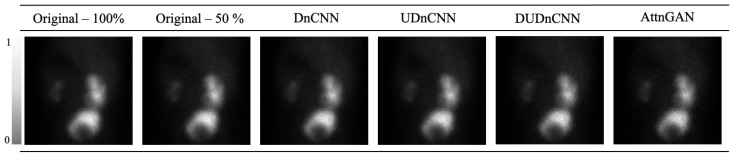
Images obtained using these optimized methods—the original (the sum of 100% of the data and the sum of 50% of the data) and with noise reduction through the implementation of the different networks. The color scale is presented in grayscale.

A visual comparison of the original images and the denoised images processed using the various approaches is shown in Figure 8.

**Figure 9:**
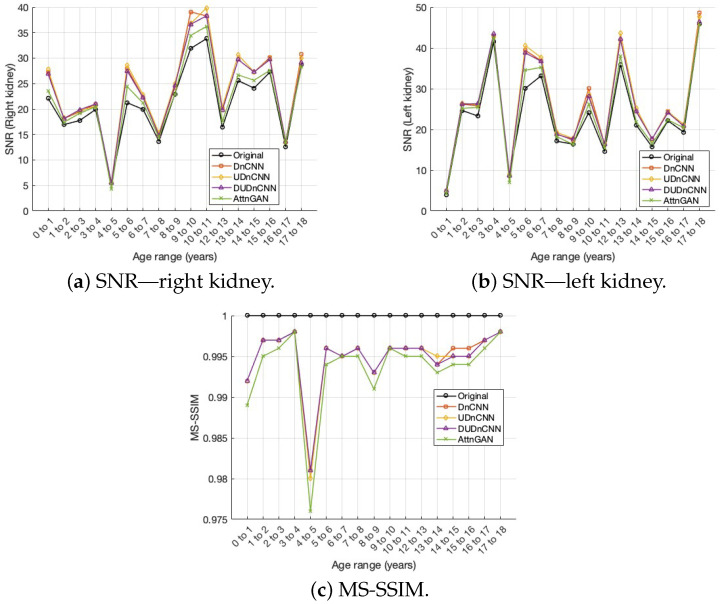
Comparison of the SNRs of the right and left kidneys and the MS-SSIMs of the different architectures.

In addition, a comparative analysis was carried out to evaluate the performances of the different neural networks applied to noise reduction in pediatric scintigraphic images, considering different age groups, as can be seen in Figure 9.

The *p*-values for the statistical analyses of the right kidney’s SNR, left kidney’s SNR, and MS-SSIM difference for the various processing methods are presented in Table 5, Table 6 and Table 7, respectively.

The training that provided the best performance (UDnCNN) required a training time of approximately 5 h. Table 8 shows the training and inference times for all the neural networks.

## 4. Discussion

This work investigates the abilities of four neural networks to denoise pediatric renal scintigraphic images. The primary task involved denoising using both the original data and the data obtained from summing 50% of the original images.

The dose reduction was simulated by decreasing the number of images added to each study, which increased noise levels because of the reduced information available for the final image formation.

Four neural networks were implemented: DnCNN, UDnCNN, DUDnCNN, and AttnGAN. The training progress of the implemented networks is shown in Figure 6. We trained the models across multiple epochs and observed that by epoch 1000, the variation between the epochs was less than 4%. This indicated stable performance, and we, therefore, selected 1000 epochs as the optimal number for training.

The training configurations for all the networks are shown in Table 2, detailing each combination tested. After evaluating these combinations, we identified the optimal parameters, presented in Table 3. The results show that although the batch size varied significantly across the networks, the best learning rate and optimizer remained consistent for all the networks. These parameters were adjusted to maximize the image quality while balancing structural detail preservation and noise reduction. This careful analysis allowed for identifying the configurations that optimized each model’s performance.

Figure 7 displays three box plots comparing the SNR of the right kidney, the SNR of the left kidney, and the MS-SSIM across four image sets: the original and those processed using the DnCNN, UDnCNN, DUDnCNN, and AttnGAN. The graphs indicate that the median and maximum values are consistently higher in the UDnCNN implementation.

To achieve a qualitative assessment, we compared the numerical values of the SNR and MS-SSIM between the original and preprocessed images. Table 4 provides a summary of this analysis. Through this comparison, we identified the UDnCNN network as the top performer, demonstrating the optimal balance between noise reduction and preservation of structural details. This is reflected in the variations observed: an 11.5% increase in the SNR for the right kidney, an 11.4% increase for the left kidney, and only a −0.5% change in the MS-SSIM, indicating the minimal compromise in the structural similarity.

These findings reinforce the stability and effectiveness of the UDnCNN in denoising without significant loss of detail, suggesting its strong potential for clinical applications.

Figure 8 shows a visual comparison of the original images and those processed using the different approaches. Although significant visible differences are not apparent among the processed images, they exhibit notable reductions in noise compared to that of the original image.

The analyses of the neural networks implemented highlighted differences in the performances of the SNR and MS-SSIM metrics, depending on the age groups evaluated. It was observed that the UDnCNN maintained higher SNR values for both kidneys and preserved the structures of the images (MS-SSIM) more consistently at all ages, even under reduced dose conditions. This stability is particularly important in younger children, where the smaller size of the organs and greater sensitivity to noise pose additional challenges. In comparison, the DnCNN and DUDnCNN showed greater variability in results between age groups, with lower performance in younger age groups. Although the AttnGAN showed competitive MS-SSIM values, its SNR performance was less consistent in younger patients, indicating less robustness under more challenging conditions. The results in Figure 9 reinforce the clinical application potential of the UDnCNN, showing its ability to balance noise reduction and preservation of structural details, regardless of the patient’s age.

Statistical analysis revealed significant differences (*p* < 0.05) in the kidney SNRs and MS-SSIMs across the processes (original Image, DnCNN, UDnCNN, DUDnCNN, and AttnGAN), as shown in Table 5, Table 6 and Table 7. However, these differences may not be easily discernible in clinical interpretation. This is supported by Figure 7 and Figure 8, which present the metrics and visually similar results side by side.

The training and inference times in Table 8 are indicative, varying with the available computing power. In relative terms, the existing networks (the DnCNN, DUDnCNN, and AttnGAN) had the longest times. The UDnCNN model struck the best balance between the SNR and MS-SSIM while also being the fastest. Given the importance of the inference time in clinical applications, it is noteworthy that the AttnGAN processed unseen images faster than the other networks in this study. This efficiency makes the AttnGAN a promising candidate for future research involving more complex training. However, the strong denoising results of the UDnCNN in pediatric renal scintigraphy suggest further exploration of such architectures.

Previous studies on deep-learning-based denoising in medical imaging have reported improvements in image quality. Minarik et al. [23] applied convolutional neural networks (CNNs) to whole-body bone scintigraphy, achieving a 92% noise reduction and outperforming traditional filters. However, their study focused on whole-body imaging rather than specific-organ imaging, such as pediatric renal scintigraphy. Compared to these studies, our work specifically targets pediatric renal scintigraphy and demonstrates that the UDnCNN achieves SNR improvements of 11.% for the right kidney and 11.4% for the left kidney, with a minimal MS-SSIM reduction of 0.5%. These results highlight the effectiveness of the UDnCNN in balancing noise reduction and structural preservation in this imaging context while addressing the limitations of previous studies by leveraging a publicly available dataset for enhanced reproducibility.

Direct comparisons between literature values and those obtained in this study are challenging because of varying databases and methodologies. The training data differ significantly in characteristics because of the distinct denoising tasks. Some studies focused on small animal images, while others analyzed different medical imaging techniques. Nonetheless, the results from our study (maximum SNRs of 11.5% for the right kidney and 11.4% for the left kidney, with an MS-SSIM reduction of only 0.5%) are competitive with those in the existing literature.

## 5. Limitations and Future Research

### 5.1. Limitations

This study had several limitations. The lack of data on the average radiopharmaceutical activity injected into patients limited the ability to accurately assess the impact of administered activity on the image quality. Without this information, direct and rigorous comparisons with other studies are challenging, potentially restricting the generalization of these findings. Additionally, for a given acquisition time, a gamma camera with higher sensitivity would have produced images with a higher number of counts, resulting in improved image quality. This makes it difficult to compare different datasets when imaging equipment and acquisition protocols are not identical.

Another limitation is that all the images were acquired using the same gamma camera, meaning that the variability across the different imaging systems was not considered. In clinical practice, where different devices may be used, the image quality can vary, which limits the applicability of these results to other settings. Furthermore, although quantitative metrics, such as the SNR and MS-SSIM, were used to evaluate the quality of the processed images, no qualitative assessment was performed by clinical experts. The input of nuclear medicine physicians or other healthcare professionals would be crucial to verify whether the denoised images retain their diagnostic value.

Moreover, this study did not implement a formal validation strategy, such as cross-validation or hold-out validation, to assess the reliability of the results. Although the models were trained and tested on independent image sets, a more structured validation approach could improve confidence in the robustness and generalizability of these findings. Future studies should incorporate appropriate validation methods to better evaluate the models’ performances across different datasets and in different clinical scenarios.

Finally, the approach used to simulate the reduced administered activity was based on summing partial data from the original images, allowing for a controlled assessment of its impact. However, this method may not fully replicate real clinical conditions, where patient variability and radiopharmaceutical distribution dynamics can influence the results. As a result, simply reducing the data noise may not accurately reflect low-activity scenarios in clinical practice, potentially limiting the direct applicability of these findings.

### 5.2. Future Research

Although this study demonstrated promising results, several avenues for future research remain. Expanding the dataset to include pediatric renal scintigraphic images from multiple centers, acquired with different gamma cameras and clinical protocols, would enhance the generalization and robustness of deep-learning models. Additionally, beyond the DnCNN, UDnCNN, DUDnCNN, and AttnGAN models explored in this study, future work could investigate alternative noise reduction techniques, such as wavelet-based methods or more advanced deep-learning architectures.

Another important direction would be to move beyond simulated dose reductions and investigate real-world strategies for minimizing the administered activity. This could involve acquiring low-dose images from realistic phantoms and training deep-learning models to handle various noise levels. Incorporating images obtained with different administered activities into the training process could lead to more flexible models, better suited for clinical applications.

Furthermore, a qualitative evaluation by nuclear medicine specialists would provide crucial insights into the clinical relevance of denoised images, ensuring that improved image quality translates to enhanced diagnostic accuracy. Finally, integrating these models into routine clinical workflows could optimize the diagnostic image quality, improving efficiency and precision in renal function assessments, particularly in cases where balancing radiation safety and image clarity is critical.

## 6. Conclusions

Deep-learning-based denoising techniques have demonstrated strong potential in medical imaging, particularly in reducing noise while preserving diagnostic features. However, their applications to pediatric renal scintigraphy remains underexplored, limiting direct comparisons with existing noise reduction methods.

This study applied four deep convolutional neural networks (DnCNN, UDnCNN, DUDnCNN, and AttnGAN) to a publicly available pediatric renal scintigraphy dataset. The objective was to evaluate whether deep-learning-based image enhancement could enable the acquisition of lower-dose images while maintaining clinically relevant image quality. The results indicate that the renal scintigraphic images reconstructed from a simulated 50% dose reduction retained diagnostic value, with the UDnCNN offering the best tradeoff between image quality and computational efficiency.

A comparison with related studies (Table 9) highlights the diversity of dose reduction strategies across different imaging modalities. Hsiao et al. explored clinical thresholds for dose reduction in pediatric renal scintigraphy, finding that a 50% reduction was feasible with noise correction. Minarik et al. demonstrated the effectiveness of CNN-based denoising in bone scintigraphy, achieving substantial noise reduction. Our study complements these findings by systematically evaluating multiple CNN architectures in renal scintigraphy, reinforcing the feasibility of deep learning in dose reduction strategies.

Although these findings are promising, future research should explore model generalization across different datasets and validate clinical applicability through expert evaluation. Expanding the dataset to include multi-center acquisitions and incorporating hybrid noise models could further enhance the robustness of deep-learning-based denoising in nuclear medicine imaging.

## Figures and Tables

**Table 1:** Summary of the image dataset used in this study.

Parameter	Total (100)
Gender (M/F)	65/35
Image resolution	128 × 128 pixels
Frames per study	120
Time per frame	10 s
Total duration	20 min
Training set	67%
Testing set	17%
Validation set	16%

**Table 2:** Neural network training configurations. Each training varied in terms of the hyperparameters, such as the size of the batch, the learning rate, and the optimizer, based on the error of the SNRs of the right and left kidneys and the MS-SSIM.

Training	Batch Size	Learning Rate	Optimizer
Training 1	32	$1.0\times 10^{-4}$	Adam
Training 2	32	$1.0\times 10^{-3}$	Adam
Training 3	32	$1.0\times 10^{-5}$	Adam
Training 4	16	$1.0\times 10^{-3}$	Adam
Training 5	4	$1.0\times 10^{-3}$	Adam
Training 6	(4–32) *	($1.0\times 10^{-5}$–$1.0\times 10^{-3}$) *	SGD

* Variable depending on the network.

**Table 3:** The optimized hyperparameters of the neural networks.

Neural Network	Batch Size	Learning Rate	Optimizer
DnCNN	16	$1.0\times 10^{-3}$	Adam
UDnCNN	16	$1.0\times 10^{-3}$	Adam
DUDnCNN	4	$1.0\times 10^{-3}$	Adam
AttnGAN	32	$1.0\times 10^{-3}$	Adam

**Table 4:** Comparison of the mean relative errors of the SNRs of the right and left kidneys and the MS-SSIMs among the different neural networks. (+) indicates a gain and (−), a loss.

Metric	Mean Relative Error (%)			
	DnCNN	UDnCNN	DUDnCNN	AttnGAN
SNR—Right Kidney	+10.8	+11.5	+10.1	+3.4
SNR—Left Kidney	+10.6	+11.4	+10.0	+3.4
MS-SSIM	−0.5	−0.5	−0.5	−0.6

**Table 5:** The *p*-values for the differences between the SNR values of the right kidney, obtained with the original image and the four denoising methods.

Valor-p	Original	DnCNN	UDnCNN	DUDnCNN	AttnGAN
Original		**<0.001**	**<0.001**	**<0.001**	**0.001**
DnCNN			0.449	0.125	**<0.001**
UDnCNN				**0.003**	**<0.001**
DUDnCNN					**<0.001**
AttnGAN					
For *p*-values of <0.05 (in bold), there is a significant difference.					

**Table 6:** The *p*-values for the differences between the SNR values of the left kidney, obtained with the original image and the four denoising methods.

Valor-p	Original	DnCNN	UDnCNN	DUDnCNN	AttnGAN
Original		**<0.001**	**<0.001**	**<0.001**	**0.005**
DnCNN			0.252	0.210	**<0.001**
UDnCNN				**0.013**	**<0.001**
DUDnCNN					**<0.001**
AttnGAN					
For *p*-values of <0.05 (in bold), there is a significant difference.					

**Table 7:** The *p*-values for the differences between the MS-SSIM values obtained with the original image and the four denoising methods.

Valor-p	Original	DnCNN	UDnCNN	DUDnCNN	AttnGAN
Original		**<0.001**	**<0.001**	**<0.001**	**<0.001**
DnCNN			0.317	0.157	**0.001**
UDnCNN				1.000	**0.001**
DUDnCNN					**0.001**
AttnGAN					
For *p*-values of <0.05 (in bold), there is a significant difference.					

**Table 8:** Training times, in hours, needed for the networks with optimized hyperparameters, and mean inference times (in seconds) needed to denoise the images.

Neural Network	Training Time (h)	Inference Time (s)
DnCNN	12.2	0.1328
UDnCNN	5.0	0.0791
DUDnCNN	13.2	0.1209
AttnGAN	13.3	0.0657

**Table 9:** Comparison of Dose Reduction and Noise Reduction Methods in Medical Imaging Studies [19,20,22,23].

Study	Imaging Modality	Dose Reduction	Noise Reduction Method	Key Finding
[19]	Pediatric Renal Scintigraphy	Up to 90%	Physician-Reviewed Filtering	50% dose reduction feasible with noise correction
[20]	Pediatric CT	Variable	Iterative Reconstruction	Maintained diagnostic quality despite increased noise
[22]	Pediatric X-ray	50%	S-Vue™ Scoring System	Image quality remained satisfactory
[23]	Bone Scintigraphy	50% (scan time halved)	CNN-Based Denoising	Reduced noise by 92%, superior to traditional filters
This study	Pediatric Renal Scintigraphy	50%	CNN-Based Denoising	The UDnCNN provided the best balance between quality and efficiency

## Data Availability

The data that support the findings of this study are available from an open-source database—*The database of dynamic renal scintigraphy*.

## References

[1] Kaur R., Juneja M. (2018). Comparison of Different Renal Imaging Modalities: An Overview. Prog. Intell. Comput. Tech. Theory Pract. Appl..

[2] Viteri B., Calle-Toro J.S., Furth S.L., Darge K., Hartung E.A., Otero H.J. (2020). State-of-the-Art Renal Imaging in Children. Pediatrics.

[3] Caroli A., Remuzzi A., Lerman L.O. (2021). Basic Principles and New Advances in Kidney Imaging. Kidney Int..

[4] Blaufox M.D., De Palma D., Taylor A., Szabo Z., Prigent A., Samal M., Li Y., Santos A., Testanera G., Tulchinsky M. (2018). The SNMMI and EANM practice guideline for renal scintigraphy in adults. Eur. J. Nucl. Med. Mol. Imaging.

[5] Zhang C., Schwartz M., Küstner T., Martirosian P., Seith F. (2022). Multiparametric Functional MRI of the Kidney: Current State and Future Trends with Deep Learning Approaches. RoFo Fortschritte Geb. RöNtgenstrahlen Nukl..

[6] Drubach L.A. (2017). Nuclear Medicine Techniques in Pediatric Bone Imaging. Semin. Nucl. Med..

[7] Dias A.H., Andersen K.F., Fosbøl M.Ø., Gormsen L.C., Andersen F.L., Munk O.L. (2025). Long Axial Field-of-View PET/CT: New Opportunities for Pediatric Imaging. Semin. Nucl. Med..

[8] Maliborski A., Zegadło A., Placzyńska M., Sopińska M., Lichosik M., Jobs K. (2018). The Role of Modern Diagnostic Imaging in Diagnosing and Differentiating Kidney Diseases in Children. Dev. Period Med..

[9] Conway J.J. (2007). Quo Vadis Pediatric Nuclear Medicine. Semin. Nucl. Med..

[10] Harambat J., van Stralen K.J., Kim J.J., Tizard E.J. (2012). Epidemiology of chronic kidney disease in children. Pediatr. Nephrol..

[11] Dahal S., Budoff M.J. (2019). Low-dose ionizing radiation and cancer risk: Not so easy to tell. Quant. Imaging Med. Surg..

[12] Brower C.H., Rehani M.M. (2021). Radiation risk issues in recurrent imaging. Br. J. Radiol..

[13] Martin C.J., Barnard M. (2021). How much should we be concerned about cumulative effective doses in medical imaging?. J. Radiol. Prot..

[14] Marcu L.G., Chau M., Bezak E. (2021). How much is too much? Systematic review of cumulative doses from radiological imaging and the risk of cancer in children and young adults. Crit. Rev. Oncol..

[15] Fahey F.H., Treves S.T. (2018). Standardization of Administered Activities in Pediatric Nuclear Medicine. J. Am. Coll. Radiol..

[16] Barone S., Pagliuso S., Pagliuso L. (2021). Nuclear Medicine in the Pediatric Field. J. Adv. Health Care.

[17] Society of Nuclear Medicine and Molecular Imaging (SNMMI) 2024 Update of the North American Consensus Guidelines for Pediatric Administered Radiopharmaceutical Activities. Acedido em 19 de Setembro de 2024. https://snmmi.org/Web/Clinical-Practice/Procedure-Standards/Standards/2024-Update-of-the-North-American-Consensus-Guidelines-for-Pediatric-Administered-Radiopharmaceutica.Mobile.aspx.

[18] Brady S.L., Trout A.T., Somasundaram E., Anton C.G., Li Y., Dillman J.R. (2021). Improving image quality and reducing radiation dose for pediatric CT by using deep learning reconstruction. Radiology.

[19] Hsiao E.M., Cao X., Zurakowski D., Zukotynski K.A., Drubach L.A., Grant F.D., Yahil A., Vija A.H., Davis R.T., Fahey F.H. (2011). Reduction in Radiation Dose in Mercaptoacetyltriglycerine Renography with Enhanced Planar Processing. Radiology.

[20] Nagayama Y., Oda S., Nakaura T., Tsuji A., Urata J., Furusawa M., Utsunomiya D., Funama Y., Kidoh M., Yamashita Y. (2018). Radiation Dose Reduction at Pediatric CT: Use of Low Tube Voltage and Iterative Reconstruction. Radio Graph..

[21] Miglioretti D.L., Johnson E., Williams A., Greenlee R.T., Weinmann S., Solberg L.I., Feigelson H.S., Roblin D., Flynn M.J., Vanneman N. (2013). The Use of Computed Tomography in Pediatrics and the Associated Radiation Exposure and Estimated Cancer Risk. JAMA Pediatr..

[22] Feghali J.A., Chambers G., Delépierre J., Chapeliere S., Mannes I., Adamsbaum C. (2021). New image quality and dose reduction technique for pediatric digital radiography. Diagn. Interv. Imaging.

[23] Minarik D., Enqvist O., Trägårdh E. (2020). Denoising of Scintillation Camera Images Using a Deep Convolutional Neural Network: A Monte Carlo Simulation Approach. J. Nucl. Med..

[24] DynamicRenalStudy Database of Dynamic Renal Scintigraphy. https://dynamicrenalstudy.org/browse.

[25] Gordon I., Piepsz A., Sixt R., Auspices of Paediatric Committee of European Association of Nuclear Medicine (2011). Guidelines for standard and diuretic renogram in children. Eur. J. Nucl. Med. Mol. Imaging.

[26] Stanley H., Narayanan S., Viswanath P., Bhooshan V. (2023). Satellite and Aerial Image Restoration Using Deep Reinforcement Learning. Fluct. Noise Lett..

[27] Sun J., Yang B.-H., Li C.-Y., Du Y., Liu Y.-H., Wu T.-H., Mok G.S.P. (2023). Fast Myocardial Perfusion SPECT Denoising Using an Attention-Guided Generative Adversarial Network. Front. Med..

